# Edible ectomycorrhizal fungi and Cistaceae. A study on compatibility and fungal ecological strategies

**DOI:** 10.1371/journal.pone.0226849

**Published:** 2019-12-23

**Authors:** Rui Albuquerque-Martins, Pedro Carvalho, Daniel Miranda, Maria Teresa Gonçalves, António Portugal

**Affiliations:** Centre for Functional Ecology, Department of Life Sciences, University of Coimbra, Coimbra, Portugal; University of Perugia, ITALY

## Abstract

Wild edible mycorrhizal mushrooms are among the most appreciated and prized mushrooms in the world. Despite the cultivation of ectomycorrhizal (ECM) mushrooms has been a growing subject of study worldwide, it has been hampered by the mutualistic lifestyle of the fungi. Although not being obligate symbionts, most of the species of ECM mushrooms only produce fruit bodies in association with trees or shrubs. In the present study, we aimed at understanding certain aspects of the ecology of four different edible ECM fungi: *Lactarius deliciosus*, *Tricholoma equestre*, *T*. *portentosum* and *Boletus fragrans*. Despite having a broad distribution worldwide, these fungi inhabit also Mediterranean habitats with understories typically dominated by rockroses (Cistaceae). Studying the ecology of these mutualistic fungi as well as the interaction with these species of shrubs is not only scientifically relevant but also pivotal for the discovery of profitable cultivation protocols. We evaluated the compatibility of these ECM species with five species within Cistaceae family - *Cistus ladanifer*, *C*. *psilosepalus*, *C*. *salviifolius*, *Halimium halimifolium* and *Tuberaria lignosa*. Each species of fungi proved to be able to establish mycorrhizas with at least 2 different plants species but varied in their host range of the tested Cistaceae. The dissimilarity in terms of host specificity between some fungal species seemed to be connected with the phylogenetic distances of the fungi. A correlation between the colonization percentage of the root systems and the mycelial growth rates in pure culture was found. The connection of these traits might be an important key to understanding the ecological competitor-colonizer tradeoffs of these ECM fungal species. Altogether, our study reports unknown plant-fungi combinations with economical relevance and also adds new insights about the ecology of these species of ECM fungi.

## Introduction

Edible ectomycorrhizal (ECM) mushrooms are widely appreciated for their gastronomic, nutritional and medicinal proprieties [1]. There is thereby a major interest in their cultivation worldwide.

Ectomycorrhizal symbiosis is described to be a mutualistic plant–fungus association formed between fine roots of plants and a fungus, mainly ascomycetes and basidiomycetes. This association plays a fundamental role in the ecosystems, affecting the biology, ecology, and growth of forest trees and shrubs. In this association with mutual benefits for both partners, the fungus confers protection from root diseases and helps the plant with water and nutrient absorption, while the plant provides shelter and carbohydrates to its fungal partner (reviewed in [2]).

Although many studies have been conducted trying to understand ectomycorrhizal associations at several levels in the last years, not much is still known about mycorrhizal compatibility. Some fungi are known to interact with several species of plants and having a broad host range while other species are relatively host-specific [3]. However, plants and fungi that are not reported to be associated in nature can establish mycorrhizas in laboratory conditions [4]. This phenomenon is not yet properly understood. However, it is known that the absence of exogenous glucose in mycorrhization media/substrate is fundamental to test real affinity/compatibility between two possible partners [5]. Moreover, the reduction of glucose concentration is frequently used in mycorrhizal synthesis experiments, stimulating the association (reviewed in [2]).

The position of fungi in ecological ectomycorrhizal successions in ecosystems has been studied and classified according to different features. A former classification proposed was the division of ECM fungi in two groups according to the temporal appearance of their sporocarps following tree establishment. Species in which the sporocarp appear within the first four years after planting were considered early-stage while the ones that appear in later years were considered as late-stage [6,7]. Other classifications followed considering other features such as the ability to produce mycelial strands or the colonization on root systems by secondary infection [8]. Nowadays is generally accepted that an early-stage species is adapted to rapidly colonize their host being replaced by other species–late-stage species—with competitor strategies that would be dominant over the first ones [9].

The competition between ECM fungi in the soil can be direct, so-called interference competition, or can be indirect through the depletion of resources also known as exploitation competition [10]. Higher growth rates can give a competitive advantage to fungi namely fungi with exploitation strategies. In fact, ECM fungi with higher growth rates have shown to have higher root colonization percentages [11].

The ECM edible species *Tricholoma equestre* (L.) P. Kumm., *Tricholoma portentosum* (Fr.) Quél., *Lactarius deliciosus* (L.) Gray and *Boletus fragrans* (*Lanmaoa fragrans*) Vittad. are considered as a delicacy and used in local gastronomy of many cultures [12–14]. In spite of the toxicity suspicions around *T*. *equestre*, this species was recently reported not to be toxic [15]. *L*. *deliciosus* is known to be a species with broad host range as well as is considered an early-stage fungi [16] being commonly associated with conifers. Not much is known about the range of hosts and the position of *T*. *equestre* and *T*. *portentosum* in ecological successions although the phylogenetically close species *T*. *matsutake* is reported to be a late-stage and dominant ECM species [17] being found associated with conifers. The same lack of knowledge is noted about the ecology of *B*. *fragrans*. However much more is known about the ecology of other species from *Boletus* genus, such as *Boletus edulis* which is a late-stage species [16] with a broad host range [18,19]. As other boletes, *B*. *fragrans* is known to be found in broadleaf forests generally associated with Oaks.

Cistaceae is a Mediterranean family comprising 8 different genera and almost 200 species (reviewed in [20]). All Cistaceae are able to establish ectomycorrhizas with a wide range of fungal taxa (reviewed in [21]). Additionally, all Cistaceae can establish arbuscular mycorrhizas in initial life stages (reviewed in [2]). *Cistus* and *Halimium* are two of the most widespread Cistaceae genera and known to be commonly associated with some ectomycorrhizal mushrooms [22], including some edible species [19,23–26]. Furthermore, there are some studies describing the synthesis and cultivation of ECM edible mushrooms (*Terfezia* spp., *Tuber* spp., and *Boletus* spp.) using several species of *Cistus* (Cistaceae) as plant partners [23,25,27–30]. In spite of *Tuberaria* genus has been much less studied, it is known that some species establish ectomycorrhizas with edible *Terfezia* [31,32].

In the present work, we aimed to:

1. Evaluate the compatibility of four species of edible ECM fungi from three different phylogenetic families (Tricholomataceae, Russulaceae, and Boletaceae)—*T*. *equestre*, *T*. *portentosum*, *L*. *deliciosus*, and *B*. *fragrans–*with five species from Cistaceae,—*C*. *ladanifer*, *C*. *salviifolius*, *C*. *psilosepalus*, *H*. *halimifolium*, and *T*. *lignosa—*a family known to have a broad range of ECM symbionts;
2. Evaluate the colonization strategies of each one of these fungi;
3. Understand how wide/narrow is the range of the tested Cistaceae hosts for each fungus.

## Materials and methods

### Fungal material

Mycelial cultures were isolated from fresh sporocarps of the four edible ECM species, *Tricholoma equestre*, *Tricholoma portentosum*, *Lactarius deliciosus and Boletus fragrans*. All sporocarps were collected in *Pinus pinaster* or *Quercus* spp. woods, with *Halimium halimifolium* and *Cistus* spp. understory in Coimbra district. The collection sites, dates and the habitat of each species can be consulted in Table 1. All the work was carried out in public lands and permits were not required, since mushroom collection is not protected under any national legislation in Portugal. Morphological identification was according to different mushroom field guides (e.g. [33,34].

**Table 1:** NCBI accession number, geographic origin, collection date and habitat of the fungal species used in this study.

Species	NCBI codes	Coordinates	Collection Date	Habitat
*Tricholoma equestre*	MG334287	40°21'01.9"N 8°41'39.7"W	Oct-14	Coastal Pinewood
*Lactarius deliciosus*	MG334288	40°21'01.9"N 8°41'39.7"W	Oct-14	Coastal Pinewood
*Tricholoma portentosum*	MG334289	40°10'48.9"N 7°39'40.1"W	Oct-14	Coastal Pinewood
*Boletus fragrans*	MN314115	40°10'08.5"N 8°32'45.3"W	Nov-14	Oak forest

Sporocarps were superficially sterilized with a solution of 3% calcium hypochlorite and dissected in aseptic conditions. Small fragments of tissue were removed from the inner part of the stem and the cap and placed into Petri dishes (n = 8) with PDA medium (Difco^™^) with pH 5.8–6.3 in a growth chamber at 24ºC ± 1°C in the dark. All mycelial cultures were subcultured every three months and periodically checked for contaminations. After the establishment of pure cultures (1 month), the morphological identification was confirmed for each species at the molecular level by sequencing ITS region (rDNA). The DNA was extracted with REDExtract-N-Amp^™^ (SIGMA-ALDRICH ® Company), using 1 mm^2^ of a pure mycelium culture in 10 μL of Extract solution and submitting it to 94°C for 10 min, 60°C for 13 min and 10°C for 15 min. After that, an equal volume of Dilution solution was added. PCR was performed using 10 μL of REDExtract-N-Amp^™^ PCR ReadyMix^™^(SIGMA-ALDRICH ® Company) combined with ITS1-F/ITS4 primers [35,36] at 0.4 μM and 1 μL of DNA template in the final volume of 20 μL. Cycling was performed with the following parameters: 1 step of 95ºC for 5 min, 35 cycles of 95ºC for 45s, 56ºC for 45s and 72ºC for 1min, and 1 step of 72ºC for 10 min. Sequences were acquired by the modified Sanger method performed by STAB VIDA ©, Portugal, and edited using Geneious® software. A Basic Local Alignment Search Tool (BLAST) was performed in the National Centre for Biotechnology Information (NCBI) database to confirm species taxonomic identification. The NCBI accession numbers are presented in Table 1.

### Phylogenetic analysis

A phylogenetic analysis was performed to explore the phylogenetic relationships among the fungal species. Firstly, the sequences were aligned together with other sequences retrieved from GenBank using the MUSCLE algorithm in MEGA7® software. After using the Akaike information criterion (AIC) in jModelTest the phylogenetic analysis was performed using maximum likelihood methods with the GTR model of evolution [37,38] in Phylemon 2.0 [39] using PhyML [37]. The bootstrap likelihood ratio test with 1000 repetitions was used to assess the Branch support. The resulting tree was represented using FigTree software [40].

### Ectomycorrhizal synthesis and assessment

Seeds from *C*. *ladanifer*, *C*. *salviifolius*, *C*. *psilosepalus*, *H*. *halimifolium* and *T*. *lignosa* were collected in different locations with high ECM mushroom diversity. The geographic location, habitat and collection date of Cistaceae seeds can be found in S1 Table. Seed sterilization was performed by placing seeds inside 1,5ml microcentrifuge tubes with sterile distilled water and then exposed to 100 ºC for 10 minutes in a water bath. Afterwards, seeds were placed in Petri dishes with MS agar medium [41]. The contaminated seeds were discarded every three days for one week. Only seedlings with developed cotyledons and radicle (2mm) were used for mycorrhizal establishment.

According to the work of Duddridge [5], an agar medium without any glucose addition should be used to test the plant-fungus compatibility. Therefore, we used half-strength MS agar medium [41] without glucose for the mycorrhization studies. To sustain the growth habit of Cistaceae shrubs for extended periods we used large flasks (Ø 5.5cm) as containers. It was added 35ml of ½ MS agar medium (Duchefa Biochemie^™^) to each flask. Patches of fungal inoculum previously grown in PDA medium were placed on a ditch opened at the agar medium. Afterwards, a sterilized aluminium disk was placed upon the culture medium, to protect both roots and fungus against photo-oxidation, and hollows were made for each of the three seedlings placed per flask immediately after the fungal inoculation. An aluminium cover was also added outside the flask at the height of the agar, coating both fungus and roots. Three flasks were used for each plant-fungus combination (n = 3). All flasks were aseptically closed and placed in a growth chamber at 20 ºC ± 1 for 5–6 months under fluorescent light (110 μmol s-1 m-2 [400–700 nm], 16 h/day).

Plant roots were regularly checked for ECM establishment (*in-situ* macroscopic observation). After 5–6 months, the ectomycorrhizal root tips were removed, washed in distilled water, examined and photographed under a stereomicroscope. The background of the ectomycorrhizal root tips’ photographs was removed using Adobe Photoshop CS5 program, Adobe Systems, Inc. ©. Morphological characterization scoring colour, shape, ramification type, ramification order and abundance was performed according to Agerer and Rambold [42]. To check for the presence of the mantle and Hartig net, the putative ectomycorrhizal root tips were cleared making some modifications in the protocol described by Phillips and Hayman [43]. Root tips were cleared with a KOH solution (10%) at 20–25 ºC for 3–5 hours, rinsed in tap water for 5 minutes and afterwards acidified with HCl solution (20%) for 1 hour for subsequent microscopic observation. The fungal taxonomic identity of the mycorrhizae was confirmed by PCR and sequencing using the protocol described above. The DNA extraction of the ECM samples was performed using 1 mm^3^ of tissue, as described previously. However, 20 μL of Extract and Dilution solutions was added instead of 10 μL.

### Similarity analysis

To analyse the similarity of the hosts’ community, we used the Bray-Curtis index for all the pairwise combinations of fungal species and obtained a cladogram. The index and cladogram were obtained using the R [44] package vegan [45].

### ECM fungal Colonization

The colonized and non-colonized root tips were scored in 3 different plants from each combination (n = 3). The ECM colonization percentage (CP) were calculated based on the following formula CP = [((nº colonized root tips)/(total nº root tips))*100]. After testing the data homoscedasticity and normality, separate One-way ANOVA were used to test if the colonization percentages were statistically different for each fungus with different plants and for each plant with different fungi using the R package car [46]. The data was plotted using the R package ggplot2 [47].

### Fungal growth rates in pure culture

To understand the ecological colonization strategies of these species of ECM fungi and to validate their growth in MS medium, the mycelial growth rates in pure culture were studied. The mycelial growth was determined in ½ MS agar and also in biotin-aneurine-folic acid (BAF) [48] medium. BAF medium was used as a control due to be a culture medium widely used for mycorrhizal synthesis. For each treatment, squares with 0.25cm^2^ were transferred from pure cultures (in PDA) and placed in the centre of 6 cm ᴓ Petri dishes (n = 5) of each culture medium. Colonies growth was measured every 3 days for 36 days by delimiting the mycelium´s area with a permanent marker at the bottom of the Petri dish. The area was calculated with the aid of Adobe Photoshop CS5 program, Adobe Systems, Inc. ©. Growth rates (cm/day) were determined for every 3 days using the following formula: GR = [((Final growth)-(Initial growth))/3]. The final growth rate for each replicate was determined by the average of the growth rates of each time point. After testing the data homoscedasticity and normality, a one-way ANOVA was used to test the significant differences between the culture media for each fungal species using the R car package [46]. The bar plots were created using the R package ggplot2 [47].

Morphological descriptions of the mycelium were made scoring several features as “Mycelium texture”, “Mycelium colour”, “Border”, “Border colour”, “Reverse colour” and “Density”.

### Correlation analysis

To analyse if the fungal growth rates correlate with the colonization percentages, we calculated the Pearson correlation coefficient using the fungal growth rates of each fungus in MS medium against the mean of the colonization percentages of the same fungus in all plant species altogether. It was not possible to perform this test per plant species due to some plants only established mycorrhizal association with 2 species of fungi (n<3). After testing the data homoscedasticity and normality, an ANOVA was used to test the statistical significance of the correlation. This analysis was done using the Analysis ToolPak from Microsoft Excel.

## Results

### ECM fungi identification

The taxonomic identity of the ECM fungi was confirmed using ITS barcoding region (Table 1). A Phylogenetic tree was made using several voucher sequences of representative species from each taxonomic group (S1 Fig). A sequence of *Tuber melanosporum*, an Ascomycota, was used as outgroup. We could observe a major division in two phylogenetic clades separating the *Boletus* from *Tricholoma* and *Lactarius* or mushrooms with pores from mushrooms with gills, respectively. Moreover, a subclade dividing *Tricholoma* from *Lactarius* was also observed.

### Fungal ECM compatibility with Cistaceae

**Fig 1:**
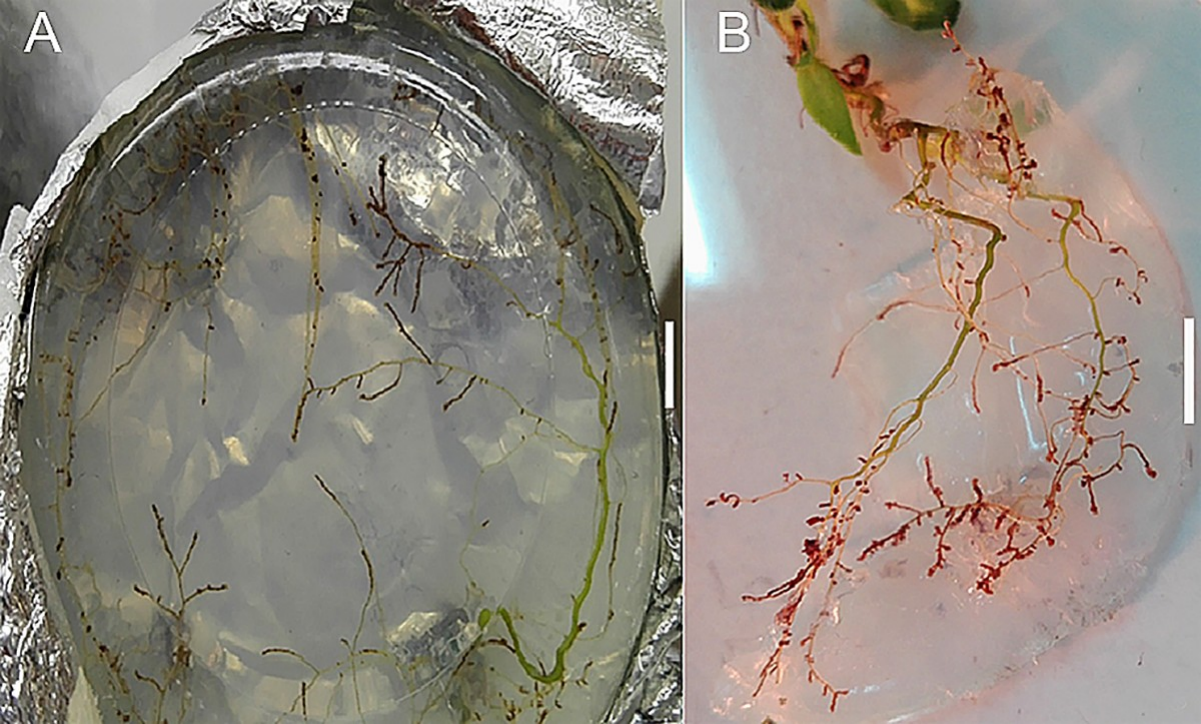
Example of ectomycorrhizal colonization (*C*. *psilosepalus* + *L*. *deliciosus*) of the root system after (**A**) two months (observed through the agar); and (**B**) 5–6 months (after removal from the agar medium) (Bar = 1cm).

During the experiment, both plants and fungi had suitable growth, never becoming senescent. The first putative ectomycorrhizal root tips were observed 2 months after inoculation (Fig 1A). Those were solitary and scattered, becoming much more abundant, dense and spread along the entire root system after 5–6 months (Fig 1B) in almost all the combinations.

All fungi were compatible with at least two species of Cistaceae. A summary of the success of each combination plant-fungus can be seen in Table 2.

**Table 2:** Ectomycorrhizal establishment between the four species of fungi and five species of Cistaceae. “+”/“-” successful/unsuccessful establishment of ectomycorrhizas.

Species	*C*. *psilosepalus*	*C*. *salviifolius*	*C*. *ladanifer*	*H*. *halimifolium*	*T*. *lignosa*
*L*. *deliciosus*	⁺	⁺	⁺	⁺	⁺
*T*. *equestre*	⁻	⁻	⁺	⁺	⁻
*T*. *portentosum*	⁺	⁻	⁺	⁺	⁺
*B*. *fragrans*	⁺	⁺	⁻	⁻	⁻

**Fig 2:**
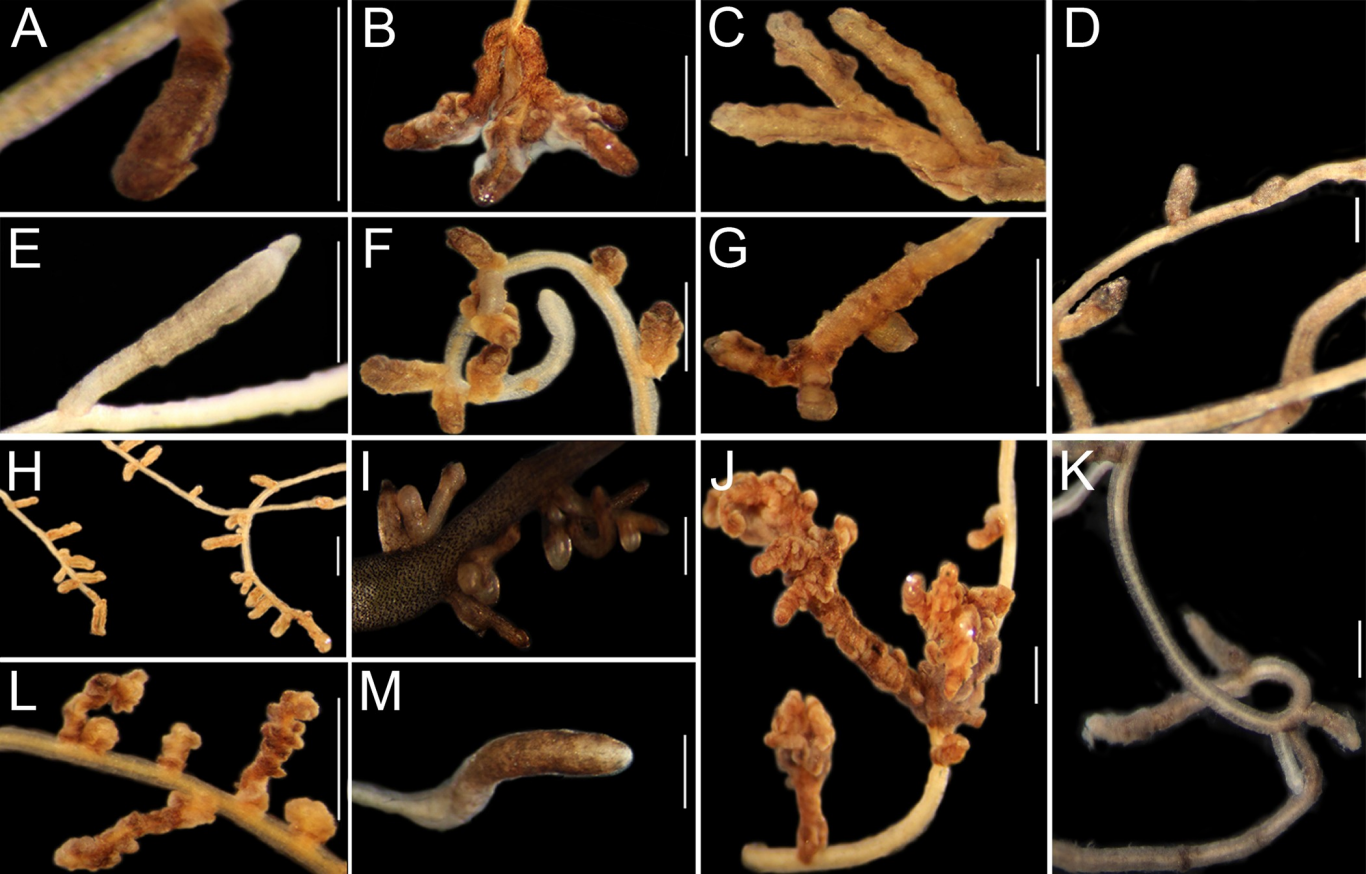
Ectomycorrhizal root tips of: *T*. *portentosum* with *C*. *psilosepalus* (**A**), *C*. *ladanifer* (**B**), *H*. *halimifolium* (**C**) and *T*. *lignosa* (**E**); *T*. *equestre* with *C*. *ladanifer* (**F**) and *H*. *halimifolium* (**G**); *L*. *deliciosus* with *C*. *ladanifer* (**H**), *C*. *salviifolius* (**I**), *C*. *psilosepalus* (**J**), *H*. *halimifolium* (**L**) and *T*. *lignosa* (**M**); and *B*. *fragrans* with *C*. *psilosepalus* (**D**) and *C*. *salviifolius* (**K**). (Bar = 1mm).

Successful mycorrhization was observed in several plant-fungus combinations with the exceptions of *Tricholoma equestre* with *C*. *psilosepalus*, *C*. *salviifolius* and *T*. *lignosa*; *Boletus fragrans* with *C*. *ladanifer*, *H*. *halimifolium* and *T*. *lignosa* and *T*. *portentosum* with *C*. *salviifolius*. The ectomycorrhizal root tips, of each combination, are presented in (Fig 2A–2M). The number of Cistaceae hosts were higher for *Lactarius deliciosus* (five species) followed by *Tricholoma portentosum* (four species). The species with a lower range of hosts were *B*. *fragrans* and *T*. *equestre* being only associated with 2 species each.

Morphologically, the colour, shape, ramification type, ramification order and abundance varied even within the combinations with the same fungal species (Table 3). The morphology of *T*. *equestre* and *B*. *fragrans* morphotypes almost did not vary with different plant partners, unlike the other fungal species.

**Table 3:** Morphological description of ECM root tips from different plant-fungi combinations.

Morphotype	Colour	Shape	Ramification type	Ramification order	Abundance
*T*. *portentosum* + *C*. *psilosepalus*	brown	straight	absent	1	solitary or in small numbers
*T*. *portentosum* + *C*. *ladanifer*	brownish	beaded	dichotomous	2	abundant, dense
*T*. *portentosum* + *H*. *halimifolium*	yellowish	straight	absent	1	abundant, dense
*T*. *portentosum* + *T*. *lignosa*	greyish	constricted between older and younger parts	absent	1	solitary or in small numbers
*T*. *equestre* + *C*. *ladanifer*	yellowish brown	straight	absent	1	abundant, dense
*T*. *equestre* + *H*. *halimifolium*	yellowish brown	constricted between older and younger parts	absent	1	abundant, dense
*L*. *deliciosus* + *C*. *ladanifer*	yellow	straight	absent	1	abundant, dense
*L*. *deliciosus* + *C*. *salviifolius*	yellowish	constricted between older and younger parts	absent	1	abundant, dense
*L*. *deliciosus* + *C*. *psilosepalus*	brownish	beaded	monopodial-pinnate	3	abundant, dense
*L*. *deliciosus* + *H*. *halimifolium*	brown	beaded	absent	1	abundant, dense
*L*. *deliciosus* + *T*. *lignosa*	brown	straight	absent	1	solitary or in small numbers
*B*. *fragrans + C*. *psilosepalus*	brown	straight	absent	1	solitary or in small numbers
*B*. *fragrans + C*. *salviifolius*	brown	straight	absent	1	solitary or in small numbers

Anatomically, it was observed an uncommon winding mantle structure in the majority of the ECM root tips, clearly visible in Fig 2B, 2J and 2L. In all the successful combinations, the presence of the mantle and the Hartig net was confirmed in cleared roots, indicating the establishment of a functional association (as example, see S2 Fig). However, it was not possible to classify the type of mantle organization of the mycorrhizas formed by each fungal species due to this atypical thin and winding mantle structure. Neither rhizomorphs nor emanating hyphae were observed.

### Similarity Analysis

**Fig 3:**
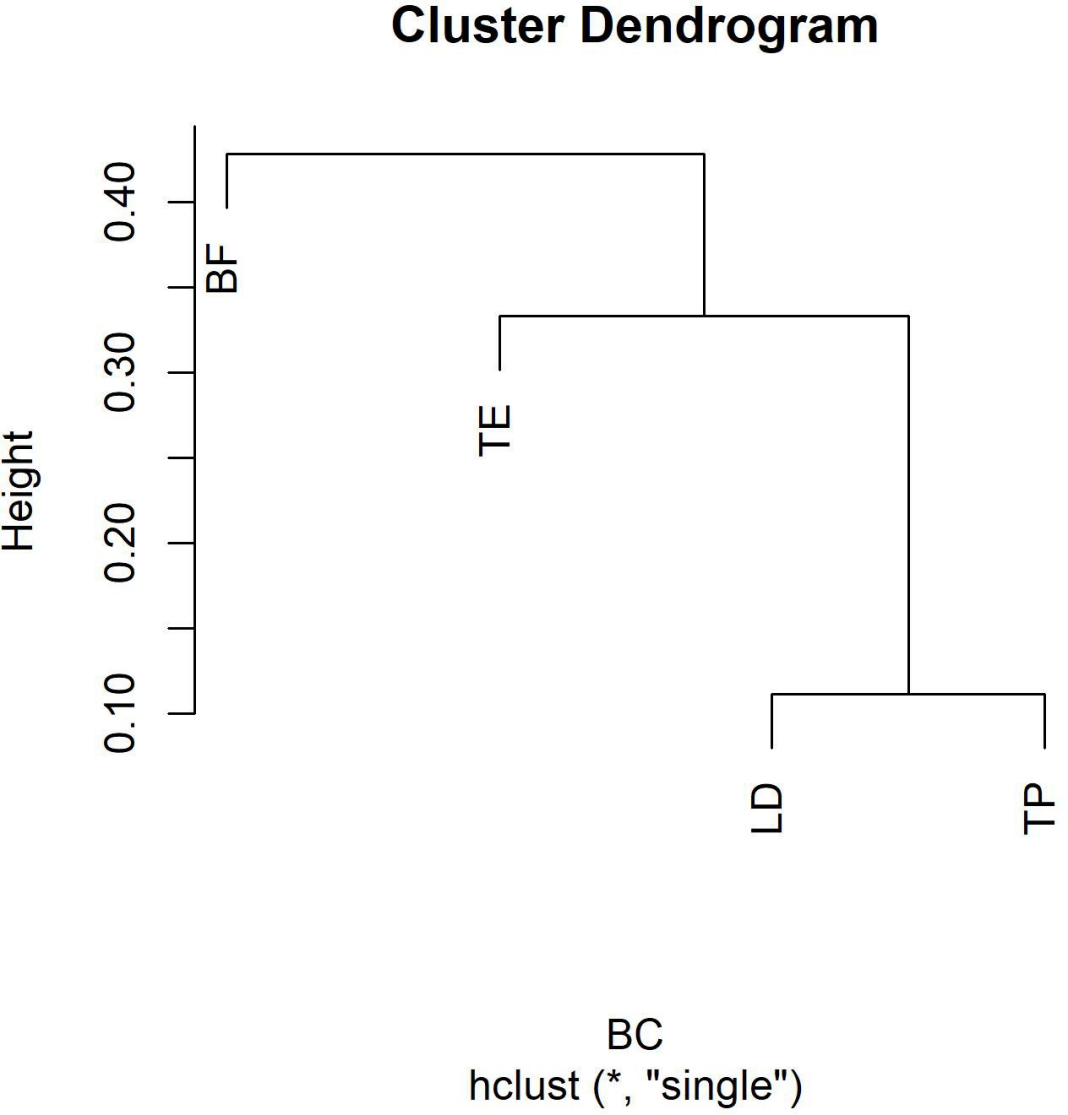
Cluster dendrogram using “single” agglomeration method, obtained based on Bryan-Curtis dissimilarity index matrix. The *y*-axis demonstrates a rescaled distance cluster combinations. (BF–*Boletus fragrans*, TE–*Tricholoma equestre*, TP–*Tricholoma portentosum* and LD–*Lactarius deliciosus*).

The highest similarity values were found between *L*. *deliciosus* and *T*. *portentosum*, which was expected since they associate with almost the same species of Cistaceae being *C*. *salviifolius* the only exception. The lowest value (0) was found for *T*. *equestre* and *B*. *fragrans* since they do not colonize any common host among the studied Cistaceae. When plotted, it can be observed an expected cluster between *T*. *portentosum* and *L*. *deliciosus*. A secondary one can also be found between *T*. *equestre* and these two species, with *B*. *fragrans* as the most dissimilar species. (Table 4, Fig 3).

**Table 4:** Similarity matrix calculated using the Bryan-Curtis index of dissimilarity between the different fungal species according to the ability of establishing ectomycorrhizae with the different tested Cistaceae species.

* *	*Boletus fragrans*	*Lactarius deliciosus*	*Tricholoma equestre*
*Lactarius deliciosus*	0.5714286		
*Tricholoma equestre*	0,00	0,5714286	
*Tricholoma portentosum*	0,33	0,8888889	0.3333333

### Root colonization percentages

**Fig 4:**
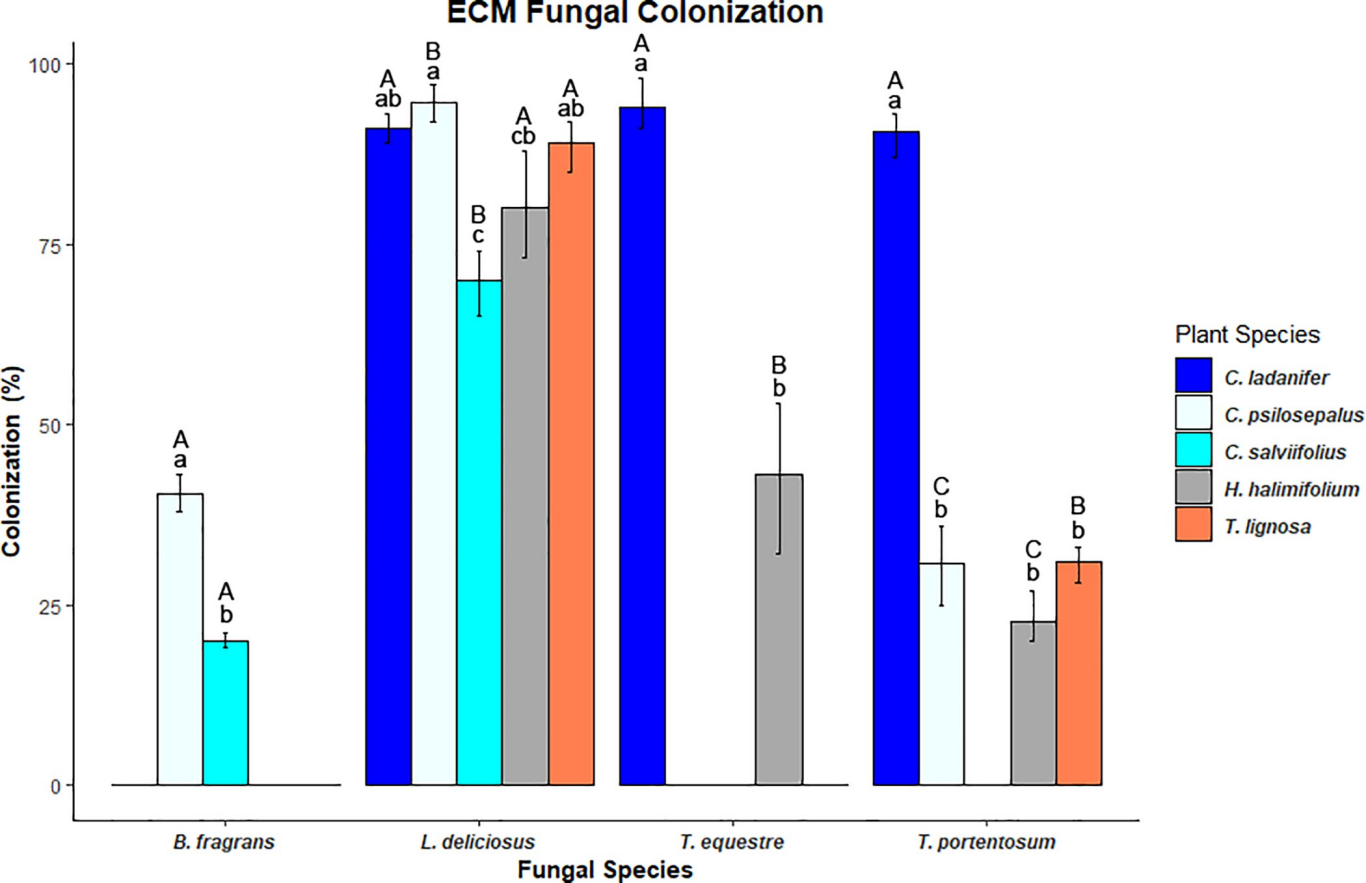
Root colonization percentages of different species of Cistaceae by *T*. *equestre*, *T*. *portentosum*, *L*. *deliciosus*, and *B*. *fragrans*. Lower case letters state for statistical differences in the colonization of the same fungi in different plants. Upper case letters state for statistical differences in the colonization of different fungi in the same plant (P<0,05). (n = 3).

The fungi had different percentages of root system colonization in different hosts as can be observed in Fig 4 (the significance values can be consulted in S2 Table). However, some patterns were observed, namely the high colonization of *C*. *ladanifer* roots by all the studied fungi. It was also observed that *L*. *deliciosus* had the highest colonization percentages in all the hosts. The lowest root colonization percentages of *C*. *psilosepalus*, *H*. *halimifolium*, and *T*. *lignosa* were observed with *T*. *portentosum*. Moreover, the lowest root colonization percentage of *C*. *salviifolius* was observed with *B*. *fragrans*.

### Correlation of mycelial growth with root colonization

**Fig 5:**
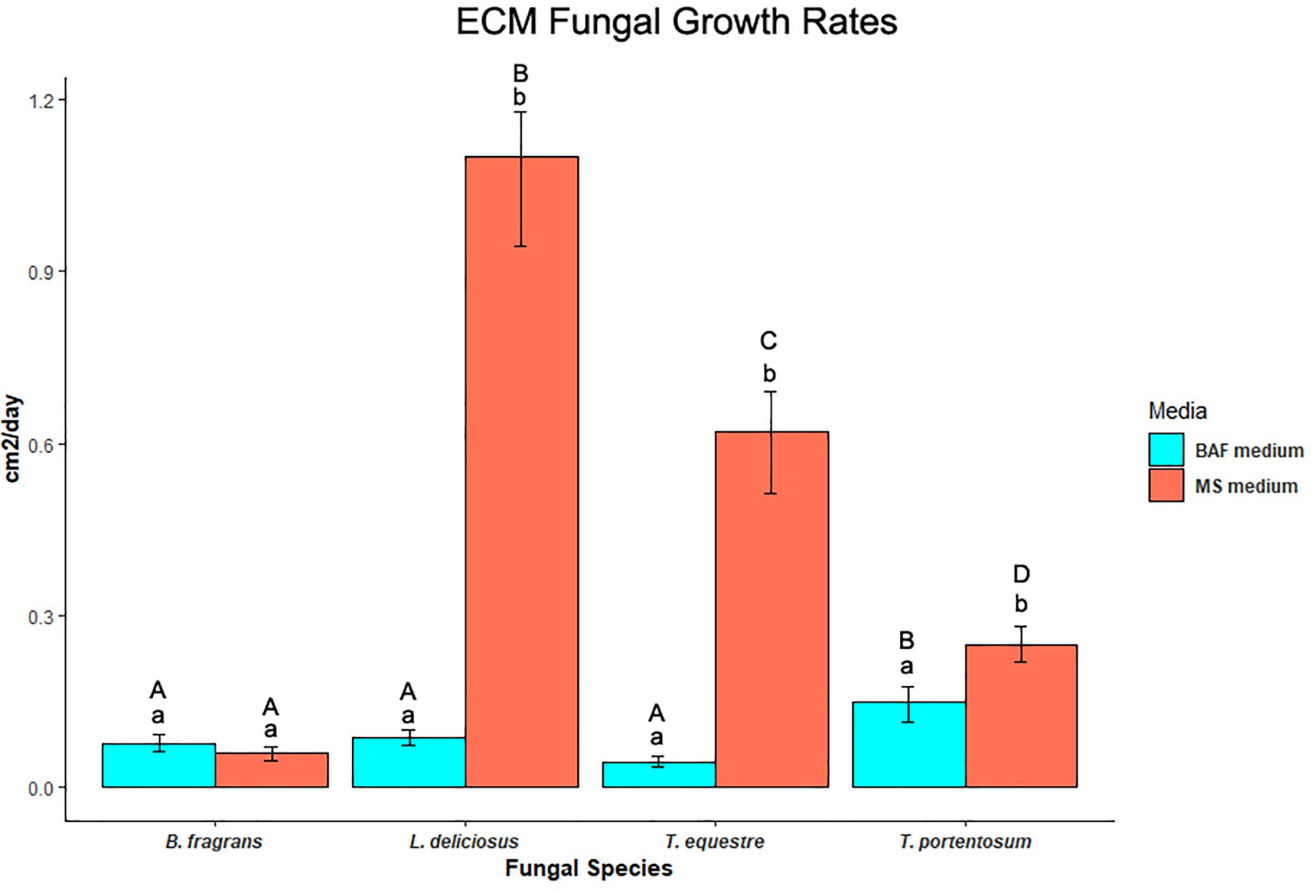
Growth rates of *T*. *equestre*, *T*. *portentosum*, *L*. *deliciosus* and *B*. *fragrans* mycelium in BAF and MS media. Lower case letters state for statistical differences in the mycelial growth rates of the same fungus in different media. Upper case letters state for statistical differences in the mycelial growth rates of different fungi in the same medium (P<0,05). (n = 5).

All the fungal species grew in both tested culture media, evidencing the suitability for fungal growth of MS, a plant-specific medium, as shown in Fig 5 (The significance values can be consulted in S3 Table). However, the fungal growth in MS medium without glucose was quite diffuse and with low hyphae density. All the mycelial morphological features of these fungi in pure culture are presented in S4 Table. The growth rates in BAF medium were generally much lower than in MS medium although the mycelium density was higher in BAF. *Boletus fragrans* had the lowest mycelial growth rates of all the fungi. *Tricholoma portentosum* had the highest growth in BAF medium whilst *L*. *deliciosus* had the highest growth in MS.

Having noticed that the pattern of mycelial growth rates did somehow match with the overall root colonization percentages, we hypothesized that these two features could be correlated. The Person correlation test gave us a strong correlation value: ***R* = 0.985** with statistical significance (***F =* 0.01451313** < 0.05), indicating that these features did correlate in our experiment.

## Discussion

The establishment of ectomycorrhizas in most of the plant-fungus combinations proved that all the studied fungi are compatible with at least two species of Cistaceae. On the other hand, the failure of ectomycorrhizal establishment in several plant-fungus combinations (*T*. *portentosum* with *C*. *salviifolius*; *T*. *equestre* with *C*. *salviifolius*, *C*. *psilosepalus* and *T*. *lignosa;* and also *B*. *fragrans* with *H*. *halimifolium*, *C*. *ladanifer* and *T*. *lignosa*) should not be conclusively interpreted, despite of both plant and fungus adequate growth and development. New experiments with different strains and ecotypes are needed to better clarify this subject, even though the sporocarps we used to obtain mycelial cultures were collected in the same habitat and, in some cases, the same area than the Cistaceae (Table 1, S1 Table). Putative mycorrhizal associations—based in aboveground sporocarps’ community—have been described between *Tricholoma* spp., *Lactarius* spp. and *Boletus* spp. with *Cistus* spp. (reviewed in [21,49–51]), but there are no reports inferring mycorrhizal establishment between these genera and *Halimium halimifolium*. In addition, several studies conducted in Spain [19,24,30] reported the ectomycorrhizal association of the edible fungi *Boletus edulis* with *C*. *ladanifer* as well as data of sporocarp productivity. The formation of ectomycorrhizas of *T*. *portentosum* and *L deliciosus* on *Tuberaria lignosa* combinations is the most surprising result, since the only known association in natural conditions is with *Terfezia* spp. [32]. Thus, the present work is the first report of *T*. *lignosa* mycorrhizas with Basidiomycetes. The successful associations here reported for the first time strengthens the described plasticity of the tested Cistaceae shrubs in establishing ectomycorrhizal associations with a wide range of fungal taxa as reviewed by Comandini et al. [21] for *Cistus* spp. and by Taudiere et al. [26] for *Halimium halimifolium*.

The tested fungal species seem to have a different range of Cistaceae hosts. *L*. *deliciosus* was the species with the broadest range of hosts (5 out of 5 tested species). This result is corroborated by the literature that states this species to be a broad host range fungus [16] known to be mainly associated with several species of Pines [52,53]. It is also known that *Tricholoma equestre* and *Tricholoma portentosum* preferentially associate with *Pinus* spp. and, therefore, their sporocarps are frequent in pinewoods. Moreover, the ectomycorrhizal associations of *T*. *equestre* and *T*. *portentosum* with *Pinus densiflora* have been reported by Yamada et al. [54,55]. We found that the range of Cistaceae hosts of *T*. *portentosum* seemed to be broader (4 different species) than those of *T*. *equestre* (2 different species), although not much is known about the range of hosts of these two species. *B*. *fragrans* was one of the species with the narrowest range of Cistaceae hosts (2 different species). In spite of the sparsity of information about the range of hosts of this species, it is known to associate with *Castanea sativa* [56] and to be naturally associated with *Quercus* spp. Moreover, the congeneric species *Boletus edulis* is known to be a broad host range fungus that associates with *Cistus ladanifer* [19,30]. Similarly to our results with *B*. *fragrans*, Águeda et al. [30] reported that *Boletus pinophilus* did not establish mycorrhizas with *C*. *ladanifer*, evidencing that congeneric species may have distinct hosts.

*Boletus fragrans* was the most dissimilar species in terms of species of hosts. As shown in S1 Fig, this species is phylogenetically separated in a different clade from the others. This separation has a morphological background since this species has sporocarps with pores whilst the other 3 species are gilled mushrooms. Another clear difference is while *L deliciosus*, *T*. *portentosum* and *T*. *equestre* are known to be preferentially associated with *Pinus* spp., boletes in general, including *B*. *fragrans*, are known to associate with *Quercus* spp. However, we cannot ignore the possibility that plant phylogenetic distances influence the ability to associate with different species/clades of fungi. Considering the phylogenetic distances between the species of Cistaceae, described by the work of Guzmán et al. [57], there seems to be no connection whatsoever, in our study, between the plants phylogeny and the species of fungi.

We found that the root colonization percentages were correlated with mycelial growth rates in pure culture. This might indicate that fungi with high growth rates can be faster colonizers of root systems giving them a clear ecological advantage. The colonization-competition trade-offs are important in the ecological successions including in mycorrhizal communities [58]. Our findings are corroborated by the work of Kennedy et al. [11] that also found that fungi with higher mycelial growth rates were faster colonizers of the root systems. In our study, *L*. *deliciosus* was the fungus with overall higher growth/colonization indicating that this species might be a colonizer in ECM ecological successions. Our results are in accordance with the work of Ortega-Martínez et al. [16] that reported this species as an early stage fungus. If we exclude the colonization of *C*. *ladanifer*, the other three fungal species had overall lower colonization/growth rates than *L*. *deliciosus*, indicating that these species could be competitors in mycorrhizal successions. Some previous works with congeneric species support our findings, reporting *T*. *matsutake* [17] and *B*. *edulis* [16] as late-stage fungi.

However, a certain host might be compatible with a fungus but not preferable. The lower colonization percentages could be due to this preference, therefore explaining the difference of colonization of the same fungus in different plant species.

Our results brought new insights about the ecology of these ECM species, although future research is needed to unveil the role of the tested fungal species in ECM communities. New plant-fungus combinations with great economical potential were also reported in this study. However, the present plant-fungus combinations were obtained in controlled and axenic conditions, which might influence further ectomycorrhizal persistence. In spite of some studies reporting the persistence of ectomycorrhizas on outplanted inoculated plants (e.g.[59,60]), most of these works are with plants inoculated in soil. Furthermore, the lack of emanating elements, the thin mantles, as well as low root colonization percentages (<50%) in some of the obtained combinations might also compromise the maintenance of the mycorrhizas in outdoor plantations. Therefore, future studies determining the ectomycorrhizal persistence on outplanted inoculated plants and the factors that induce the production of fruit bodies are also needed.

## Supporting information

S1 Fig Maximum likelihood tree based on fungal ITS sequences. Maximum likelihood tree based on ITS sequences obtained from mycelial cultures isolated from sporocarps and voucher sequences obtained from GenBank (with accession numbers included). Numbers at the nodes are values for branch support that were estimated using bootstrap likelihood ration test. The evolutionary distances are indicated by the scale bar.
(PDF)

S2 Fig Example of the ECM root tips’ mantle obtained in the present study. Microscopic observation of the anatomical structures of some ectomycorrhizal root tips cleared with a 10% KOH solution (20–25 ºC for 3–5 hours) with a subsequent acidification with 20% HCl solution (for 1 hour). A Mantle of *T*. *portentosum* with *C*. *psilosepalus*. B Laticiferous hyphae (arrow) present in the mantle of *L*. *deliciosus* with *C*. *psilosepalus*. (Bar = 10μm)
(PDF)

S1 Table Geographic location, habitat and collection date of Cistaceae seeds. (XLSX)

S2 Table Tukey multiple comparisons of the means of different fungal colonization percentages of different studied fungi and plants. (XLSX)

S3 Table Statistical analysis results of the fungal growth rates in the different culture media. (XLSX)

S4 Table Morphology of *T. equestre*, *T. portentosum*, *B. fragrans* and *L. deliciosus* mycelium in MS and BAF media. (XLSX)
