# Supplementary material for: Edible ectomycorrhizal fungi and Cistaceae. A study on compatibility and fungal ecological strategies
Source: PLoS One. 2019 Dec 23;14(12):e0226849. doi: 10.1371/journal.pone.0226849 (PMC6927596; doi:10.1371/journal.pone.0226849)
Supplement: S1 Fig — Maximum likelihood tree based on ITS sequences obtained from mycelial cultures isolated from sporocarps and voucher sequences obtained from GenBank (with accession numbers included). Numbers at the nodes are values for branch support that were estimated using bootstrap likelihood ration test. The evolutionary distances are indicated by the scale bar. (PDF) [file pone.0226849.s001.pdf]

S1 Fig. Maximum likelihood tree based on fungal ITS sequences.

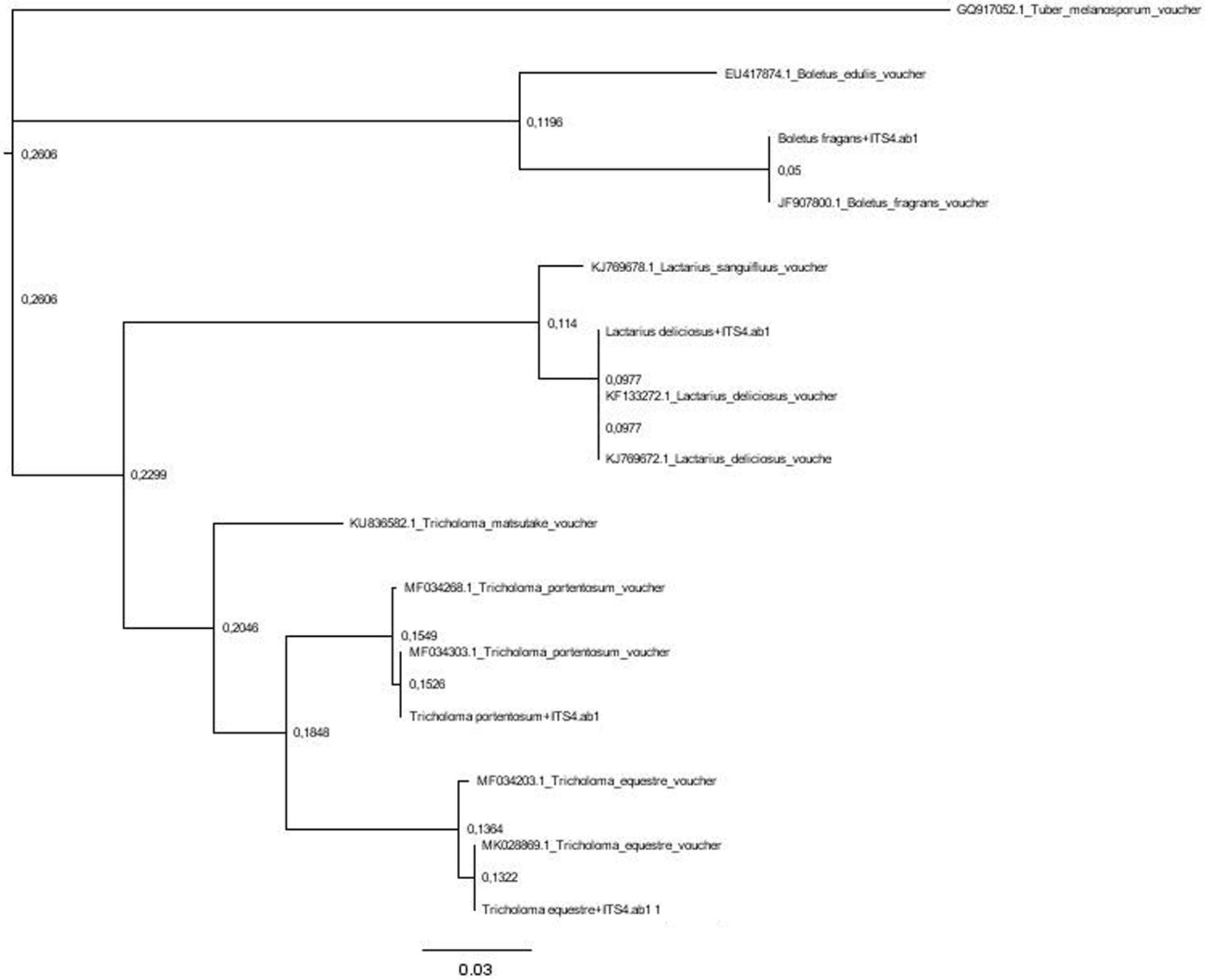

Maximum likelihood tree based on ITS sequences obtained from mycelial cultures isolated from sporocarps and voucher sequences obtained from GenBank (with accession numbers included). Numbers at the nodes are values for branch support that were estimated using bootstrap likelihood ration test. The evolutionary distances are indicated by the scale bar.
