# Supplementary material for: Edible ectomycorrhizal fungi and Cistaceae. A study on compatibility and fungal ecological strategies
Source: PLoS One. 2019 Dec 23;14(12):e0226849. doi: 10.1371/journal.pone.0226849 (PMC6927596; doi:10.1371/journal.pone.0226849)
Supplement: S2 Fig — Microscopic observation of the anatomical structures of some ectomycorrhizal root tips cleared with a 10% KOH solution (20–25 ºC for 3–5 hours) with a subsequent acidification with 20% HCl solution (for 1 hour). A Mantle of T. portentosum with C. psilosepalus. B Laticiferous hyphae (arrow) present in the mantle of L. deliciosus with C. psilosepalus. (Bar = 10μm) (PDF) [file pone.0226849.s002.pdf]

S2 Fig. Example of the ECM root tips' mantle obtained in the present study.

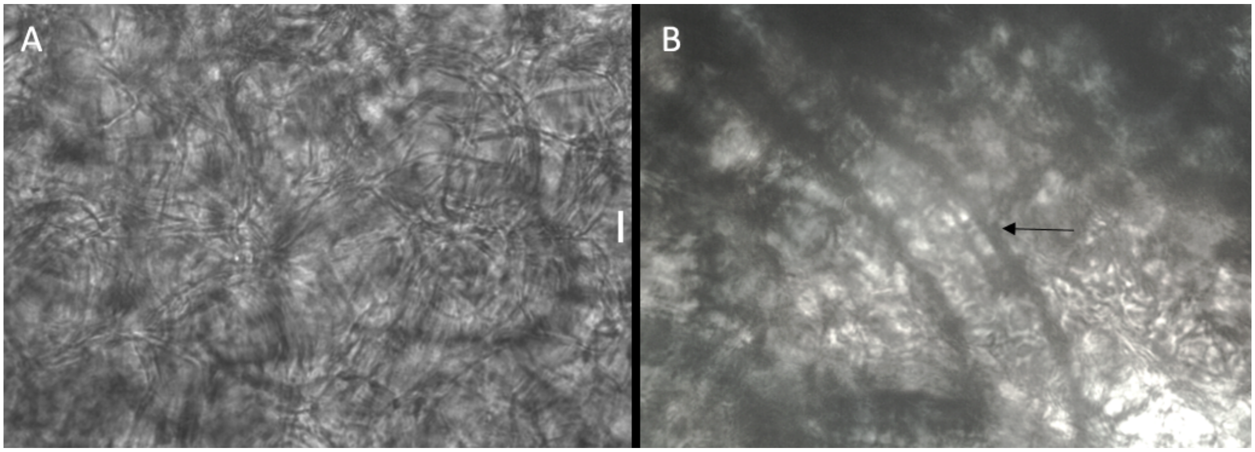

Microscopic observation of the anatomical structures of some ectomycorrhizal root tips cleared with a 10% KOH solution (20-25 ºC for 3-5 hours) with a subsequent acidification with 20% HCl solution (for 1 hour). **A** Mantle of *T. portentosum* with *C. psilosepalus*. **B** Laticiferous hyphae (arrow) present in the mantle of *L. deliciosus* with *C. psilosepalus*. (Bar= 10µm)
